# Correction to ‘The roles of T-cell competition and stochastic extinction events in CAR T-cell therapy’

**DOI:** 10.1098/rspb.2021.2786

**Published:** 2022-02-09

**Authors:** Gregory J. Kimmel, Frederick L. Locke, Philipp M. Altrock

*Proc. R. Soc. B* **288**, 20210229 (Published Online 24 March 2021) (doi:10.1098/rspb.2021.0229)

The published manuscript ‘The roles of T-cell competition and stochastic extinction events in chimaeric antigen receptor T-cell therapy’. [1] requires a correction.

For the calculation of initial tumour size, we used data from the literature, specifically as reported by Dean *et al*. [2]. We inadvertently cited the wrong source (‘[5]’ in the originally published manuscript), which might be of importance for readers in two places in our manuscript.

First, in the caption for figure 1, panel (*c*), on page 3: Median initial tumour mass was 94.86 ml; this median value originates from the combined cohort previously published in Dean *et al.* [1]. Only 22 patients of this dataset (out of 96) were on the Zuma-1 trial. All others received commercial CAR T cell therapy.

Second, in the bottom line of table 1, on page 4, for ‘initial median tumour cell number’, the reference in the last column should have cited the additional reference Dean *et al.* [2].

Of note, this error does not change any of the results or conclusions of our work. The particular tumour volume/cell number used for our modelling is not of immediate importance for our analyses, as we do not address patient-specific predictions.

Lastly, we noticed incorrectly labelled figure panels, possibly due to an error during proof stage: In figure 2, panels (*a*) and (*b*), the units on the *y*-axes should be μl^−1^, not ml^−1^.
